# The Future Prevalence of Sarcopenia in Europe: A Claim for Public Health Action

**DOI:** 10.1007/s00223-016-0220-9

**Published:** 2016-12-24

**Authors:** O. Ethgen, C. Beaudart, F. Buckinx, O. Bruyère, J. Y. Reginster

**Affiliations:** Department of Public Health, Epidemiology and Health Economics, Liège State University, Quartier Hôpital, CHU B23, Avenue Hippocrate, 13, 4000 Liège, Belgium

**Keywords:** Sarcopenia, Prevalence, Public health, Burden of disease

## Abstract

Sarcopenia is a major public health issue. To convince health policy makers of the emergency to invest in the sarcopenia field, it is of critical importance to produce reliable figures of the expected burden of sarcopenia in the coming years. Age- and gender-specific population projections were retrieved until 2045 from the Eurostat online database (28 European countries). Age- and gender-specific prevalences of sarcopenia were interpolated from a study that compared prevalence estimates according to the different diagnostic cutoffs of the EWGSOP proposed definition. The reported prevalence estimates were interpolated between 65 and 100 years. Interpolated age- and gender-specific estimates of sarcopenia prevalence were then applied to population projections until 2045. Using the definition providing the lowest prevalence estimates, the number of individuals with sarcopenia would rise in Europe from 10,869,527 in 2016 to 18,735,173 in 2045 (a 72.4% increase). This corresponds to an overall prevalence of sarcopenia in the elderly rising from 11.1% in 2016 to 12.9% in 2045. With the definition providing the highest prevalence estimates, the number of individuals with sarcopenia would rise from 19,740,527 in 2016 to 32,338,990 in 2045 (a 63.8% increase), corresponding to overall prevalence rates in the elderly of 20.2% and 22.3% for 2016 and 2045, respectively. We showed that the number of sarcopenic patients will dramatically increase in the next 30 years, making consequences of muscle wasting a major public health issue.

## Introduction

Sarcopenia is clinically defined as a progressive and generalized loss of skeletal muscle mass and strength: It is the major pathway to physical frailty [1, 2]. Until recently, sarcopenia was considered as a geriatric syndrome but it is now recognized as an independent condition by an International Classification of Disease, Tenth Revision, Clinical Modification (ICD-10-CM), code (i.e., M 62.84) [3]. Over the last decade, definitions of sarcopenia, among researchers, have varied and sometimes were discrepant [4, 5]. In 2010, the European Working Group on Sarcopenia in Older People (EWGSOP) published its recommendations for a clinical definition and consensual diagnosis criteria of sarcopenia [6], which includes a combination of a loss of muscle mass and strength or physical performance. This panel of respected experts suggested an algorithm for sarcopenia case-finding in older individuals based on measurements of gait speed, grip strength and muscle mass [6]. However, prevalence of sarcopenia remains difficult to establish. Indeed, it can differ based on the characteristics of the studied population (e.g., subjects living in nursing homes have a higher prevalence) but can also dramatically change depending on the definition used for the diagnosis of sarcopenia [7]. A major step toward obtaining a more accurate picture of sarcopenia prevalence is that, since 2010, most of the studies have used the EWGSOP consensus as the gold standard to define sarcopenia. However, within the consensual definition, different cutoff points are recommended for the diagnosis of sarcopenia [6]. Two options for each variable (skeletal muscle mass index, muscle strength and physical performance including more specifically gait speed) are suggested to define subnormal values. Subsequently, in subjects aged 65 years and older, prevalence of sarcopenia may differ from 9.25 to 18%, when the two cutoff points proposed by the EWGSOP for lean mass, muscle strength and gait speed are selected and combined [8]. Sarcopenia is now a major public health issue. It has been widely associated with negative health outcomes, including but not exhaustively physical disability, falls, injurious falls, nursing home admissions, depression, hospitalizations and mortality [9–11]. All these consequences are linked to direct healthcare costs. In 2000, these were estimated to raise up to 18.5 billion USD in the USA [8, 12]. Reducing the prevalence of sarcopenia by 10% would result in saving 1.1 billion USD per year in the USA [13]. There is no doubt, because of the current burden of sarcopenia but also because the number of older people is increasing all over the world that health policy decision-makers will soon consider financial investment in sarcopenia prevention and treatment to ensure important future savings [14]. However, to convince health authorities of the emergency to invest in the sarcopenia field, it is of critical importance to produce reliable figures of the expected burden of sarcopenia in the coming years. Therefore, we projected the potential future prevalence of sarcopenia in Europe for the next 30 years. We used age and gender-specific European population projections, and the various diagnostic cutoff points proposed by the EWGSOP, for lean mass, muscle strength and gait speed [6, 8].

## Materials and Methods

### Demographic Data

Age- and gender-specific population projections until the year 2045 were retrieved from the Eurostat online database [15]. Data were extracted for the 28 countries of the European Union on 11.01.2016, using the last updated main scenario projection available of the EUROPOP13 (08.12.2014).

### Definition of Sarcopenia

We followed the definition of sarcopenia proposed by the EWGSOP [6], which offers two thresholds for muscle mass, grip strength and gait speed, respectively [6]. These thresholds resulted from an exhaustive analysis of the existing literature dealing with the assessment of muscle mass, muscle strength and muscle function [6].

### Sarcopenia Prevalence

**Fig. 1:**
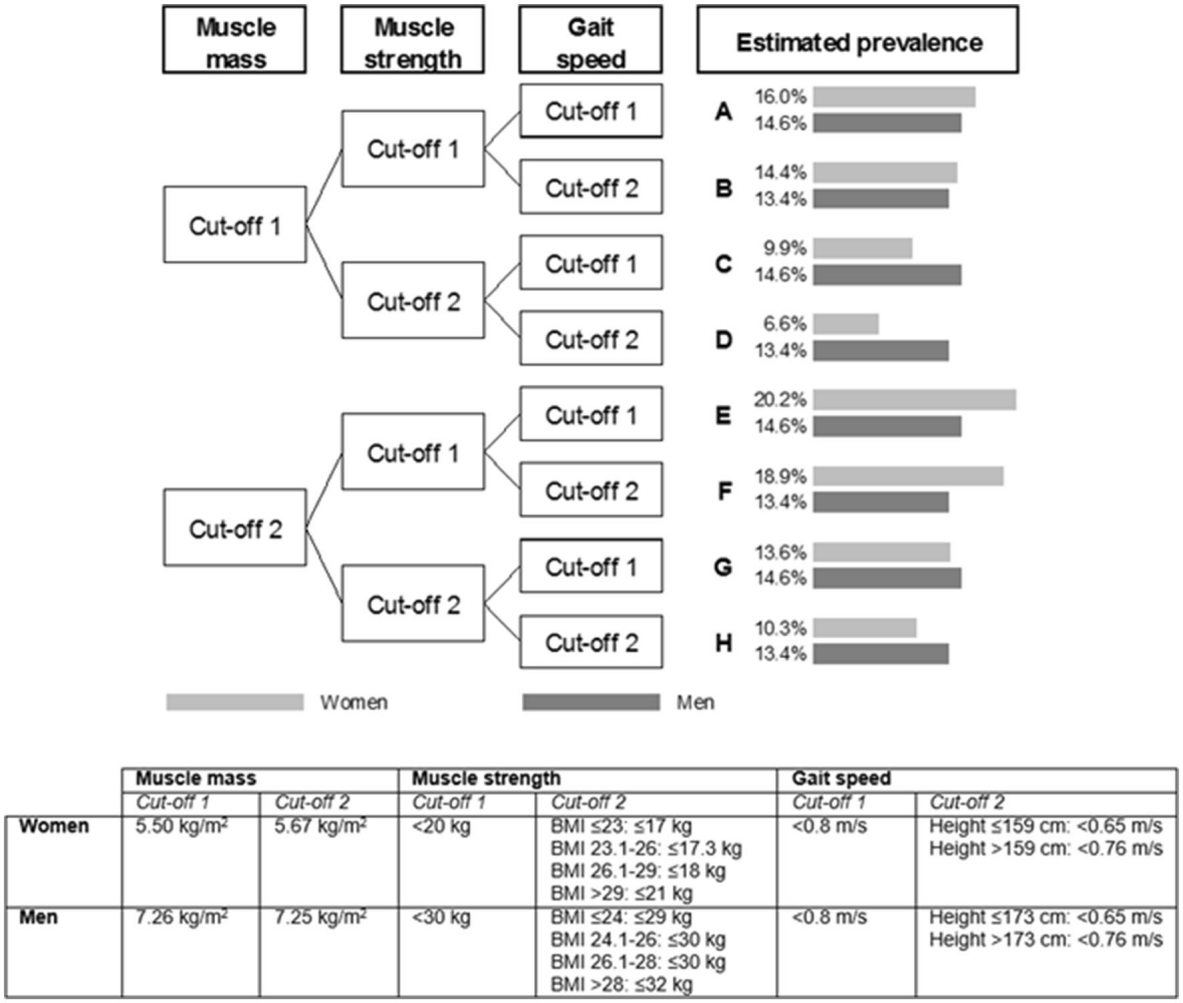
Prevalence of sarcopenia according to the different cutoffs of the EWGSOP definition

**Fig. 2:**
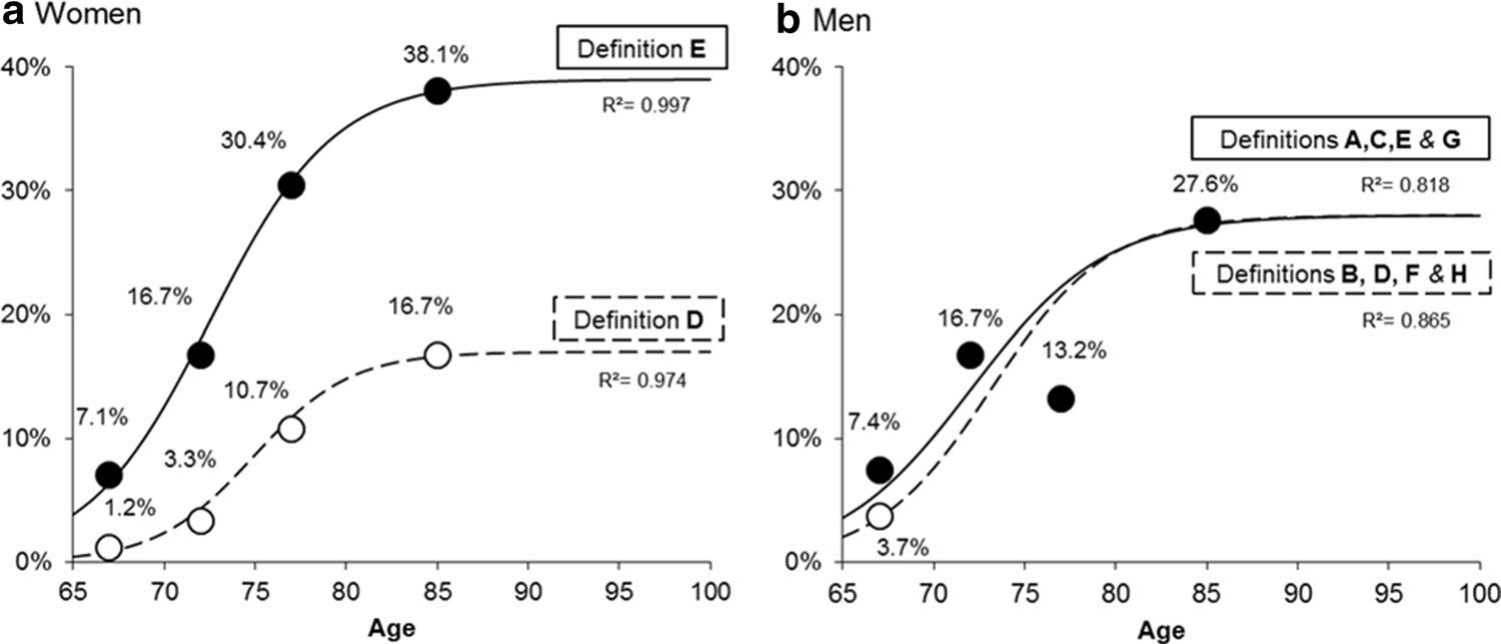
Age- and gender-specific interpolation of sarcopenia prevalence using Logistic equation

Age- and gender-specific prevalences of sarcopenia were interpolated from a study that precisely compared prevalence estimates according to the different diagnostic cutoffs of the EWGSOP proposed definition [8] (Fig. 1). Among the eight possible definitions of sarcopenia enabled by the EWGSOP (named from A to H in this manuscript), we selected for both men and women the definitions that gave the lowest (i.e., definition D for women 6.6% and definitions B, D, F, H for men 13.4%) and the highest (i.e., definition E for women 20.2% and definitions A, C, E, G for men 14.6%) prevalence estimates in order to obtain a range of prevalence projections. Figure 2 shows that in women the range of prevalence estimate is relatively broad as compared to men when there is little variation in the prevalence estimates provided by the different definitions.

### Projection Model

In the study by Beaudart et al. [8], prevalence estimates were reported by 5-year age categories from the age of 65 years (i.e., 65–69, 70–74, 75–79 and ≥ 80 years). These age-groups estimates were applied to the central age of each age category (i.e., 67, 72, 77 and 85 years, respectively). The prevalence of sarcopenia was then interpolated continuously between 65 and 100 years of age using the Logistic equation (Fig. 2):

$$P\left( t \right) = \frac{M}{{\left( {1 + M{\text{e}}^{ - \alpha t + \beta } } \right)}}$$

where *P*(*t*) is the prevalence of sarcopenia at age *t*, *M* the maximum level of prevalence possible, *α* and *β* the two coefficients of the Logistic equation. A distinct Logistic regression was fitted for men and for women and for each of the definition providing the highest and the lowest prevalence estimates in both genders (i.e., four Logistic equations were fitted in total).

A Logistic interpolation approach was preferred as it is self-constrained and explicitly considers a maximum level than cannot be exceeded (the plateau or saturation level, denoted *M*). *M* was set at 17 and 39% for definition D and E in women and at 28% for all definition in men. This approach avoids unrealistic growth (such as exponential or power) of prevalence as individual age. All interpolating Logistic equation yielded satisfactory *R* ^2^ values above 80% (Fig. 2).

Interpolated age- and gender-specific estimates of sarcopenia prevalence were then applied to the Eurostat population projections until 2045. As sensitivity analysis, all prevalence estimates reported were varied within a ±20% range and re-interpolated using the same method.

## Results

The EU28 population is expected to raise from 509,164,624 individuals in 2016 to 525,171,079 individuals in 2045 (a 3.1% increase). The proportion of women above the age of 65 years should increase from 21.1% in 2016 (54,942,878 women) to 30.0% in 2045 (80,105,255 women), i.e., a 31.4% increase The proportion of men in this age group should increase from 16.5% in 2016 (41,004,959 men) to 25.2% in 2045 (65,050,660 men), i.e., a 37.0% increase.

**Fig. 3:**
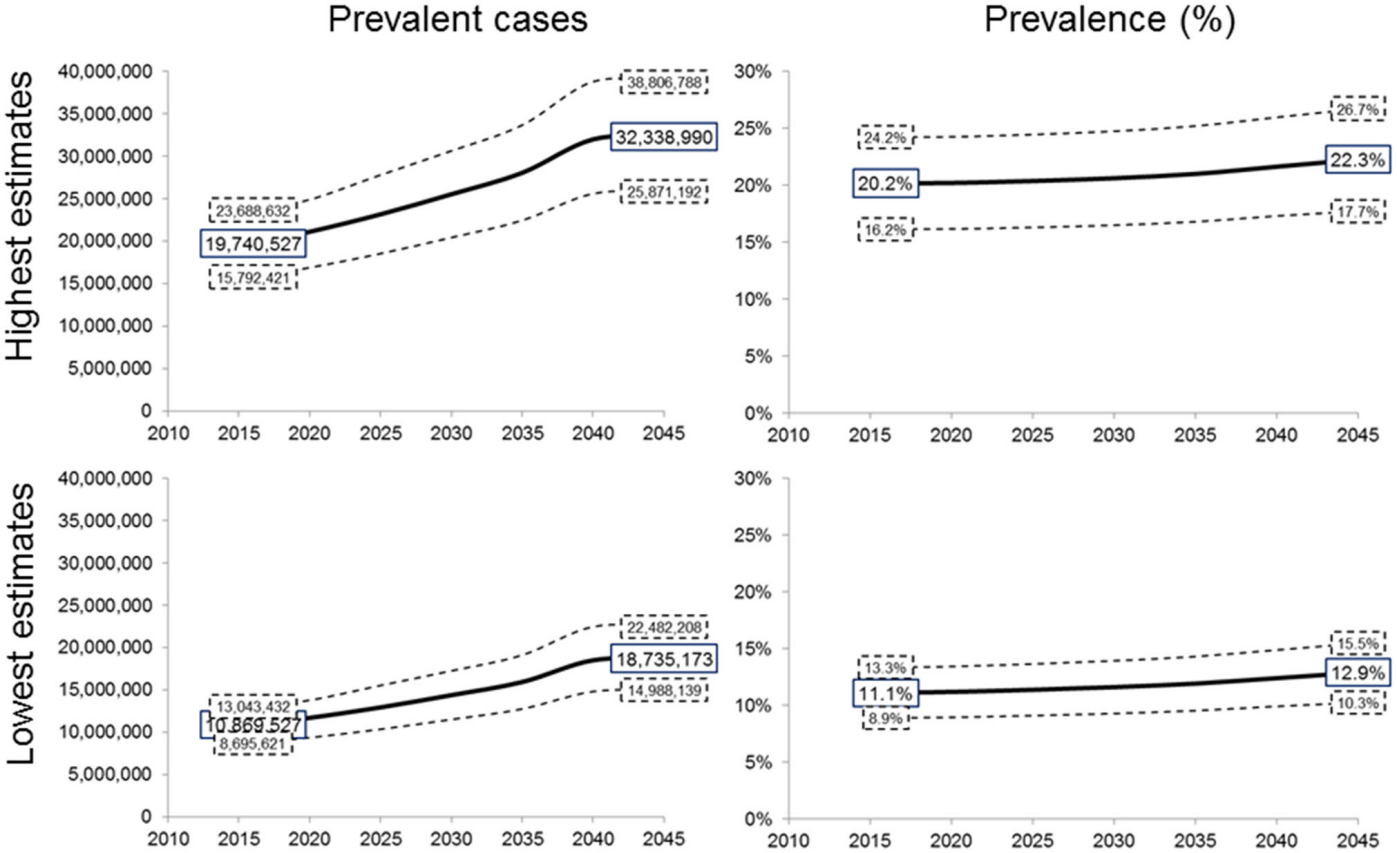
Projected prevalence of sarcopenia in Europe from 2016 to 2045 according to the highest and the lowest definition variants (*dotted boxes* and *lines* represent the ±20% sensitivity analyses)

Using the definition providing the lowest prevalence estimates, the number of individuals with sarcopenia would rise in Europe from 10,869,527 in 2016 to 18,735,173 in 2045 (a 72.4% increase). This corresponds to an overall prevalence of sarcopenia in the elderly rising from 11.1% in 2016 to 12.9% in 2045 (Fig. 3).

With the definition providing the highest prevalence estimates, the number of individuals with sarcopenia would rise from 19,740,527 in 2016 to 32,338,990 in 2045 (a 63.8% increase), corresponding to overall prevalence rates in the elderly of 20.2 and 22.3% for 2016 and 2045, respectively (Fig. 3).

**Fig. 4:**
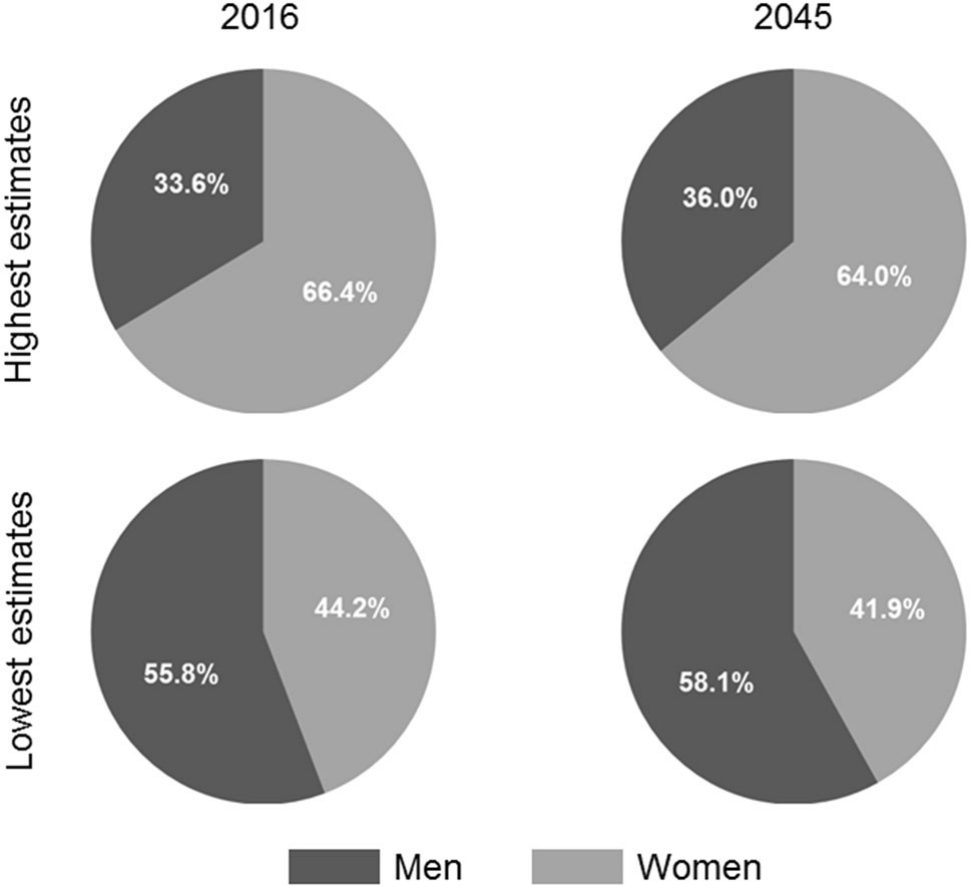
Proportion of men and women among prevalent cases of sarcopenia from 2016 to 2045 and according to the highest and the lowest definition variants

Women would account for 44.2–41.9% of prevalent cases in 2016–2045 with the lowest estimates but for 66.4–64.0% of prevalent cases in 2016–2045 with the highest estimates, respectively (Fig. 4).

## Discussion

Our results suggest that, during the next 30 years, the prevalence of sarcopenia will significantly increase, independently of the chosen definition of the condition. This increase in prevalence corresponds to a number of sarcopenic individuals varying between 18 million and 32 million, within the EU28 countries, as a function of the selected diagnostic thresholds, for muscle mass, muscle strength and gait speed.

Several factors may affect the observed prevalence of a chronic disorder. Sarcopenia being an age-related disorder, its prevalence will be influenced by the longevity of the population. Recent probabilistic projections suggest that, in the next decades, whereas the rate of increase in life expectancy will decline, gains will continue asymptomatically, at a linear rate on average [16]. Therefore, our assumption of an increase in life expectancy in both genders, between now and 2045, based on the last updated main scenario projection available for the EU seems realistic. An increased mortality rate may also negatively impact the prevalence of a chronic disorder. Whereas it is well accepted that sarcopenia is directly linked to frailty [17], and subsequently to at least a twofold increase in overall mortality compared to age-matched subjects [18], the recent recognition of sarcopenia as an independent disease [2] will most likely contribute to an earlier diagnosis and to a more dedicated care, all factors contributing to a longer life of sarcopenic patients. The attention focused on sarcopenia will also induce research and development toward an improvement of the currently available diagnostic tools [19], allowing for a wide availability of more accurate and affordable equipments, another factor that might significantly contribute to an increase in the observed prevalence of the disease, hence suggesting that our projection rates remain highly conservative. The progressive switch between bioimpedance and dual-X ray absorptiometry (DXA) for the measurement of appendicular lean mass (ALM) was indeed followed by the detection of a greater number of sarcopenic cases [20]. However, preventative measures, including nutritional optimization, food supplements or regular resistance-training physical exercise become widely recommended in elderly subjects and may improve musculoskeletal heath, reduce or delay the development of muscle wasting and subsequent sarcopenia [21–24], hence decreasing its prevalence in the coming years. Whereas several potential promising pharmacological interventions are under development to improve physical performance in elderly subjects with frailty or sarcopenia [25–27] the likelihood that these interventions will fully revert the process of loss of muscle mass, strength or performance is very low, which means that even if widely available in a soon future, these therapeutic option will not impact on the prevalence of the disease.

The figures presented in this study may be challenged because we based over assumptions on one single definition of sarcopenia, even widely accepted, i.e., the EWGSOP 2010 consensus [6]. Selecting other definitions would most likely result in different absolute numbers of projected patients [7]. Similarly, the prevalence of sarcopenia for the various thresholds of skeletal muscle mass, muscle strength and physical performance were also derived from one single study [8] and using other sources might lead to the selection of different values of baseline prevalence. However, these changes in crude figures and raw numbers of current or projected patients would not modify the global trend of our study suggesting a real twenty-first century “epidemics” of sarcopenia and frailty if appropriate attention is not promptly brought to this issue.

A potentially important issue, which might be faced in the coming years, if DXA remains the gold standard for the assessment of ALM is the impact of the growing number of obese individuals [28] on the observed prevalence of sarcopenia. Precision and accuracy of DXA for the assessment of ALM is significantly lowered in obese individuals compared to people with normal weight [29]. Increased fat mass may indeed blunt the identification of low ALM and somehow “mask” sarcopenia, which may in turn artifactually decrease the number of properly identified sarcopenic subjects.

In conclusion, by using a projection model based on the current prevalence of sarcopenia and demographic data available for the population of 28 countries of EU, we suggest that the number of sarcopenic patients will dramatically increase in the next 30 years, making consequences of muscle wasting a major public health issue. These figures can be used as a basis to motivate heath policy makers and health providers to consider muscle health as a first healthcare priority in the soon future.
